# 

**Published:** 1844-12

**Authors:** 


### WESTERN JOURNAL

OF

### MEDICINE AND SURGERY.

### EDITORS.

---

DANIEL DRAKE, M. D.,

PROFESSOR OF PATHOLOGY AND THE PRACTICE OF MEDICINE
IN THE MEDICAL INSTITUTE OF LOUISVILLE:

LUNSFORD P. YANDELL, M. D.,

PROFESSOR OF CHEMISTRY AND PHARMACY IN THE SAME:

THOMAS W. COLESCOTT, M. D.

### TO CORRESPONDENTS AND SUBSCRIBERS.

With the present number, another volume of the *Western Journal of Medicine and Surgery* is completed. In prospect of the coming year, we have no new promises to make, nor any fresh inducements to hold out to subscribers or correspondents; but we stand pledged to continue our exertions to render the Journal worthy of public patronage. The plan of the work will not be in any respect changed, but as we have the promise of contributions from more of the Professors in the Medical Institute, than have written for it during the past year, we hope to be able to give greater variety to its pages. A number of papers promised us by distant subscribers have not yet been received. We hope these promises will soon be redeemed. In behalf of the publishers, we bespeak from subscribers early remittances. Many of these are sadly in arrears. In no year, we believe, since the Journal commenced, have the receipts been so small. This is an evil which ought to be remedied at once.

### INDEX TO VOL. II.

[NEW SERIES.]

A

Age as influencing mortality in disease, 343.
Alison's outlines of pathology and practice, 30.
Anus, artificial, 490.
Arsenic in diseases of the skin, 79.
Animal Chemistry, 85.
Asthma-ipecacuan, 179.
Accidents, a life of, 182.
Andral on the blood, 123.
Alcoholic lotion, 263.
Anderson's case of white swelling, 299.
American Journal of insanity, 326.
Anatomy, human, 325.
" Cruveilhier's, 518.
" pathological, 533.
Alienation, mental, 525.
Anatomical Atlas, 339.
Anomalies of the teeth, 353.
Antagonism of phthisis and intermittent fever, 341.
Ague, epidemic in Persia, 76.
Apparatus, vocal, hysterical affections of, 75.
Antidotes of corrosive sublimate, copper, lead and arsenic, 437.
Amenorrhœa cured by electro-galvanism, 427.
Arsenic in diseases of the skin, 79.

B

Bartlett's philosophy of medicine, 506.
Belladonna in dysmenorrhœa and neuralgia, 68.
Belladonna in scarlatina, 261.
Brodie's lecture, 148.
Bell's select medical library, 182.
Buck on phthisis pulmonalis, 93.
Blood, essay on, 123.
Barry's translation of Royer-Collard's memoir, 218.
Boling's surgical cases, 280.
" on quinine in fevers, &c. 348, 455.
Brain, human, 442.
Black-Tongue, 451.
Brodie on Mercury in syphilis, 438.

C

Caldwell on chemico-physiology, 1.
" on animal chemistry, 85.
Children, temperature of, 76.
Case of vicarions menstruation, 65.
Cyclopædia of practical medicine, 45, 147, 335.
Curling on the testis, 53.
Case of dysphonia, 67.
Cases of longevity, 64.
Convulsions of children, mustard in, 66.
Cases of hysterical affection, 75.
Case of Abdomino-intestinal wound, 100.
Case of fracture of skull, 104.
Condie on diseases of children, 108.
Cataract, spotaneous cure of, 262.
Case of white swelling, 299.
Case of rupture of Uterus, 498.
Case of mental alienation, 518.
Chronic catarrhal opthalmia, 530.
Climactric disease, 531.
Classes, medical, 549.
Cruveilhier's Anatomy, 518.
Consumption, cure of, 366.
Clergy, health of, 371.
Cure of hydrocele by iodine injections, 421.
China, progress of surgery in, 420.
Calomel in diseases of the eye, 425.

Camphor, effects of, 436.
Corrosive sublimate, antidote for, 437.
Copper, antidote for, 437.
Colombo-root in vomiting, 441.
Combe on sleep in warding off insanity, 443.
Consumption, M'Dowell on, 242.
Copland's medical dictionary, 521.

D

Diagnosis of consumption, 93.
Doctors, 82.
Death of Forry, 551.
Deceptions in medicine, 535.
Dysphonia, case of, 67.
Dysmenorrhœa and neuralgia, belladonna in, 68.
Diseases of the gums and alveoli, 28.
Diseases of the skin, arsenic in, 76.
Drake's Letters, 159, 163, 176, 271, 354, 537, 545.
Drake on diseases of the south, 181.
Drake's introductory, 550.
Dictionary of practical medicine, 521
Diseases of children, Condie on, 108.
Diseases of the eye, calomel in, 425.
Diseases of the tongue, 148.
Dawson on typhus fever, 185.
Dowler on febrile caloricity, 281.
Dunglison on human health, 330.
Dunglison's practice of medicine, 91.
Druitt's surgery, 332.

E

Epidemic Ague, 76.
Erysipelas, 78.
Epperson on laryngismus stridulus, 25.
Eye, diseases of, nitrate of silver in, 67.
Enterprise, western, 91.
Electricity, Wood on, 340.
Examinations, a manual of, 339.
Examiner, Medical, and western schools, 369.
Essay on the blood, 123.
Essay on iodine, 402.
Extirpation of the spleen, 424.
Electro-galvanism in amenorrhœa, 427.
Effects of camphor, 436.
Emphysema, case of, 417.
Epilepsy, hysterical, 536.

F

Fascination, 422.
Fainting fever of Persia, 76.
Fever, epidemic typhus, 185.
Febrile caloricity, Dowler on, 281.
Fever remittent, vs. consumption, 341.
Fevers in Liberia, 265.
Flint's Druitt, 332.
Fluorine, 424.
Fracture of skull, 104.
Fever, yellow, quinine in, 158.
Fever, yellow, in Woodville, Miss., 549.
Forry, death of, 551.

G

Girdner's case of emphysema, 417.
Griffith on diseases of the lungs, 38.

H

Harrison on the nervous system, 490
Hare-lip, operation for, 77.
Headache, nervous, 78.
Henry on black-tongue, 451.
Hydriodate of potash, 78.
Hæmoptysis, nature and treatment of, 81.
Hysterical affection of vocal apparatus, 75.
Hospitals and surgeons of Paris, 248 248.
Halford, Sir Henry, 269.
Health, Dunglison on, 330.
Hare-lip, treatise on, 335.
Hydrophobia, 343.
Health of the clergy, 371.
Hydrocele, iodine injections for, 421.
Hæmorrhage, fatal, 530.

I

Iodide of potassium, 84, 267.
Ipecacuan-asthma; 179.
Insanity, American Journal of, 326
Institute, Medical, of Louisville, 184, 372, 549.
Intermittent fever, antagonism of phthisis, 341.
Influence of the market on mortality, 423.
Inflammations, quinine in, 348.

J

Justice, professional, 351.
Journal of Insanity, 326.

K

Kilpatrick's case of abdomino-intestinal wound, 100.

L

Longevity, cases of, 64.

Longevity among the poor, 65.
Louisville summer school, 184, 372.
Louisville Medical Institute, 184, 372, 549.
Life of accidents, 182.
Larynx, foreign body in, 106.
Liberia, its fevers, 280.
Lectures, medical, 279.
Lectures on midwifery, 302.
Lancet, Western, 302.
Longevity of inhabitants of Liberia, 265.
Lee's theory and practice of midwifery, 302.
Ludlow's manual of examinations, 339.
Longet on Phrenology, 350.
Loins, pain of, 434.
Letters, traveling—Dr. Drake's, 163, 176, 270, 354, 360, 445, 456, 537, 545.
Lead, antidote for, 437.

M

Menstruation, vicarious, case of, 65.
Mustard in convulsions, 66.
Medical school, Transylvania, 92.
Medicine, Dunglison's practice of, 91.
Medical Examiner and western schools, 183.
Medicine, Louisville summer school of, 184.
Medical Institute, Louisville, 184, 372, 549.
Memoir on modifying the forms of living bodies, 141.
M'Dowell on consumption, 242, 366, 463.
Medical lectures, 280.
Midwifery, Lee's lectures on, 302.
Manual of Examinations, Ludlow's, 339.
Medical Examiner and western medical schools, 369.
Medicine, summer school of, 372.
Mesmerism, therapeutic agency of, 425.
Mesmerism, triumphs of, 450.
Medicinal vegetable, new, 372.
Mortality as influenced by price of provisions, 423.
Mortality in Great Britain, 423.
Mercury and iodine compared, 428.
Mercury in syphilis, 438.
Medicine psychological, 443.
Medical student, 513.
Moreau's midwifery, 302.
Morgan's case of rupture of the uterus, 498.
Mortality at different ages, 343.
McKintosh's practice, 420.
Monomania, 527.
Magnetized steel rod, 533.
Medicine, deceptions in, 535.
Medical profession, 536.

N

Nervous headache, 78.
Neuralgia, 68, 424.
Nervous system, Harrison on, 502.
Nervous system, theory of, 502.
New medicinal vegetable, 370.

O

O'Ferrall on iodine, 402.
Operative surgery, treatise on, 51.
Operation for hare-lip, 77.
Operations in cancerous diseases, 440.
Opthalmia, treatment of, 530.
Observation on climactric disease, 531.

P

Pain of loins, 434.
Pathology of neuralgia, 424.
Practical medicine, cyclopædia of, 45.
Pancoast's operative surgery, 51.
Potash, hydriodate of, 78.
Plants, case of the crossing of, 370.
Potassium, iodide of, 84.
Pathology and practice, outlines of, 30.
Phthisis pulmonalis, Buck on, 93.
Practical medicine, cyclopædia of, 147, 355.
Principles of medicine, 520.
Pulmonary consumption, curability of, 242.
Phthisis pulmonalis, alcoholic lotion in, 263.
Pettigrew on the superstitions of medicine, 317.
Phrenology, Longet on, 350.
Partus Siccus, 351.
Professional justice, 351.
Psychological medicine, 443.
Protective influence of vaccination, 443.
Post-mortem fever, 281.

Philosophy of medicine, 506.

Q

Quackery, 441.
Quinine in yellow fever, 158.
Quinine in fevers and inflammations, 348, 465.

R

Regimen as influencing form, 141.
Restored vision, 77.
Rice grounds, 342.
Researches on post-mortem fever, 281.
Rokitansky's pathology, 522.

S

Schools of medicine, 549.
Stramonium in laryngismus stridulus, 25.
Spermatorrhœa, 70.
Sciatica, iodide of potassium in, 84, 534.
Smyley's case of fracture, 104.
Surgical cases, 218.
Stewart on hospitals and surgeons of Paris, 248.
Scarlatina, belladonna in, 261.
Superstitions of medicine, 317.
Surgery principles and practice of, 332.
Surgery, operative, 51.
Smith's anatomical atlas, 339.
Siccus, partus, 351.
Surgery in China, 420.
Spontaneous cure of phthisis, 426.
Steel rod magnetized, 535.
Skin, arsenic in diseases of, 79.

T

Temperature of children, 76.
Transylvania medical school, 92, 549.
Testis, diseases of, 53.
Therapeutics of mesmerism, 425.
Thornton on Laryngotomy, 106.
Traveling Letters, 163, 176, 270, 354, 360, 445, 456, 537, 545.
Trachea, wound of, 417.
Tumor, enormous, 257.
Teeth, anomalies of, 353.

U

Uterus, rupture of, 490.

V

Valerianate of zinc, 341.
Viper, poison of, 343.
Vicarious menstruation, case of, 65.
Vision restored, 77.
Vegetable, new, 370.

W

Western enterprise, 91.
Western medical schools, 183, 369
Western Lancet, 279.
Wilson's human anatomy, 528.
Wood on electricity, 240.

Y

Yellow fever, quinine in, 158.
Yellow fever in Woodville, 549.

Z

Zinc, valerianate of, 341.

### *Receipts of Medical Journal for November.*

| | |
|---|---|
| Dr. M. L. Reid, Hagerstown, Ind., | $15 00 |
| Dr. B. F. Richardson, Pine Bluff, Ark., | 5 00 |
| Dr. V. Peyton, Brownsville, Miss. | 10 00 |
| Dr. T. J. Pollard, Fayetteville, Ark., | 5 00 |
| Dr. J. C. Wilson, Mt. Carmel, Ky. | 1 00 |
| Dr. J, Culbertson, Vicksburg, Ky., | 5 00 |
| Dr. S. B. Robinson, Murfreesboro, Tenn., | 5 00 |
| Dr. Mauzhas, Danville, Mo., | 5 00 |
| Dr. L. D. Anderson, Flemingsburg, Ky., | 5 00 |
| Dr. S. M. Jenkins, Columbia, La., | 10 00 |

### TO SUBSCRIBERS.

We regret to find that, notwithstanding unusual expenditures upon the present volume of this Journal and its very material improvement, our subscribers have this year been more than usually remiss. We would give notice that all who may not remit promptly shall be forthwith and constantly harrassed with every form of dun until they are worried out. Their names shall appear in a black list on the back of the Journal, and, simultaneously, they shall be called upon and persecuted by duns of every kind, travelling agents, local agents, constables, sheriffs, and catchpoles. We are shamefully deprived of our dues, and we mean to have justice done us.

PRENTICE & WEISSINGER.

☞Receipt by mail, at our risk, under frank of post-master or post-paid.

---

The WESTERN JOURNAL OF MEDICINE and SURGERY is published monthly by the undersigned, at the corner of Main and Fifth streets, Louisville, at $5 per annum, payable in advance. Each number contains 96 pages, making two volumes in the year of more than 550 pages.

Contributions to its pages by the Physicians of the Valley of the Mississippi, addressed to the Editors, are respectfully solicited.

Letters on business, to be addressed, postage paid, to the publishers.

☞Postmasters, by the regulations of the Postoffice Department, will frank letters containing subscription money, and all remittances so franked are at the risk of the publishers.

*January*, 1844. PRENTICE & WEISSINGER.

